# Expression of microRNA in human retinal pigment epithelial cells following infection with *Zaire ebolavirus*

**DOI:** 10.1186/s13104-019-4671-8

**Published:** 2019-10-01

**Authors:** Genevieve F. Oliver, Ayla V. Orang, Binoy Appukuttan, Shashikanth Marri, Michael Z. Michael, Glenn A. Marsh, Justine R. Smith

**Affiliations:** 1 Flinders University College of Medicine and Public Health, Flinders Medical Centre Room 4E-431, Flinders Drive, Bedford Park, SA 5042 Australia; 2 Health and Biosecurity, Commonwealth Scientific and Industrial Research Organisation, 5 Portarlington Rd, Newcomb, VIC 3219 Australia

**Keywords:** Ebola, Filovirus, microRNA, Retina, Retinal pigment epithelium, Uveitis, *Zaire ebolavirus*

## Abstract

**Objective:**

Survivors of Ebola virus disease (EVD) are at risk of developing blinding intraocular inflammation—or uveitis—which is associated with retinal pigment epithelial (RPE) scarring and persistence of live *Zaire ebolavirus* (EBOV) within the eye. As part of a large research project aimed at defining the human RPE cell response to being infected with EBOV, this work focused on the microRNAs (miRNAs) associated with the infection.

**Results:**

Using RNA-sequencing, we detected 13 highly induced and 2 highly repressed human miRNAs in human ARPE-19 RPE cells infected with EBOV, including hsa-miR-1307-5p, hsa-miR-29b-3p and hsa-miR-33a-5p (up-regulated), and hsa-miR-3074-3p and hsa-miR-27b-5p (down-regulated). EBOV-miR-1-5p was also found in infected RPE cells. Through computational identification of putative miRNA targets, we predicted a broad range of regulatory activities, including effects on innate and adaptive immune responses, cellular metabolism, cell cycle progression, apoptosis and autophagy. The most highly-connected molecule in the miR-target network was leucine-rich repeat kinase 2, which is involved in neuroinflammation and lysosomal processing. Our findings should stimulate new studies on the impact of miRNA changes in EBOV-infected RPE cells to further understanding of intraocular viral persistence and the pathogenesis of uveitis in EVD survivors.

## Introduction

Survivors of Ebola virus disease (EVD) suffer long-term sequelae. Intraocular inflammation—termed uveitis—develops in 18–34% of survivors [1, 2], and 40% of affected persons become blind [3]. Live *Zaire ebolavirus* (EBOV) has been isolated from intraocular fluid after resolution of the viremia [4], and retinal scars that indicate involvement of the retinal pigment epithelium are associated with uveitis [3]. *Zaire ebolavirus* may persist at immune-privileged sites, such as the eye, since local immune responses are attenuated to avoid tissue damage. We previously demonstrated that human retinal pigment epithelial (RPE) cells mount a type I interferon (IFN) anti-viral response and maintain immunomodulatory activity when infected with EBOV, and this cell population may be a reservoir for EBOV in the eye [5].

MicroRNAs (miRNAs) are short (18–22 nt) fragments of single-stranded RNA that bind the 3′ untranslated region of mRNA, and directly suppress or indirectly activate gene expression. Because only a small (7–8 nt) “seed region” requires complementarity to permit binding, a single miRNA may have hundreds of mRNA targets, and thus play a complex regulatory role in cellular activity that varies by cell type and disease state [6]. We used small RNA sequencing (RNA-Seq) to identify miRNA expression in EBOV-infected human RPE cells, and conducted in silico analyses to identify biological targets of, and molecular interactions with, these miRNAs.

## Main text

### Methods

Total RNA was sourced from our previous study [5], in which the human RPE cell line (ARPE-19) [7] was infected in triplicate with EBOV (multiplicity of infection, 5) or mock-infected for 24 h. From 1 μg of RNA extract, small RNA was selectively enriched through sequential adapter ligation to 3′ and 5′ ends of RNA fragments using the TruSeq Small RNA Library Preparation Kit (Illumina, San Diego, CA). Single-stranded cDNA was synthesized by reverse transcription, and separately amplified with polymerase chain reaction using one of 48 primers containing index sequences (11 cycles). Using the Pippin Prep DNA Size Selection System (Sage Science, Beverley, MA), amplified cDNA constructs were purified from 3% agarose gel to isolate a library of small clone fragments. The library was sequenced on Illumina NextSeq 500, using NextSeq 75-cycle High Output Kits (Illumina), with the PhiX Control v3 library (Illumina) as sequencing control.

Short reads were filtered for adapters and reads of low quality using Cutadapt (v.1.8) [8] with error rate of 0.2 and minimum length of 18 bp, aligned against GENCODE human genome reference assembly GRCh38.p3 using Burrows Wheeler Aligner [9], and assigned to miRBase (v.21) annotations using HTSeq (v.0.6.1p2) [10, 11]. Data were filtered for targets with a minimum of 10 counts in at least 50% of samples. After normalization, differentially expressed miRNAs between EBOV- and mock-infected ARPE-19 cells were identified using the DESeq 2 statistical package (v.3.2) [12]. Reads were screened for complete matches to published EBOV miRNA sequences, allowing for 3 mismatches [13]. Raw data from our RNA-Seq study of the total RNA transcriptome of EBOV-infected ARPE-19 cells [5] (https://www.ncbi.nlm.nih.gov/geo/query/acc.cgi?acc=GSE100839) were processed in the same statistical pipeline used for human miRNAs, to identify genes that were differentially regulated with adjusted p-value ≤ 0.05 and log2 fold-change ≥ 1.

Computational predictions of the targets of differentially expressed human miRNAs (defined by adjusted p-value < 0.001 and log2 fold-change > 1) were performed using public databases with different algorithms: Diana microT CDS (v.5.0) [14, 15], filtering with a threshold of 0.95; TargetScan (v.7.1) [16], sorting on a total context score < 0.15; and miRDB (v.5.0) [17], with a target threshold > 85. The human miRNAs were also input into public online repositories of experimentally-validated data on molecular interactions: miRecords (release 2013) and miRTarBase (v.7.0) [18, 19], set to ‘strong evidence’.

Gene ontology [20] and pathway analyses were performed using Cytoscape (v.3.4.0) and ClueGO (v.2.3.3) plugin [21, 22], to identify enriched biological processes and molecular functions, and Kyoto Encyclopedia of Genes and Genomes (KEGG) pathways [23]. Using the STRING Action dataset (v.9) within CluePedia plugin (v.1.3.3) [24, 25] and collating miRNA-gene pairs, a miRNA-based network was constructed from miRNA-predicted gene target lists, miRNA-validated target gene lists, and the differentially expressed gene list; interactions were demonstrated based on degree, to identify the most highly-connected genes and miRNAs. An inverse correlation was required between expression of an miRNA and its target gene in these analyses.

### Results

RNA sequencing yielded 1.61–2.87 × 10^7^ reads per replicate (Additional file 1). Filtering for size, 91–97% of trimmed sequences were mapped to the human genome. 814 human miRNAs were identified (Additional file 2), and multidimensional scaling showed separation between results for EBOV- and mock-infected human RPE cells (Additional file 3). Defining differential expression as adjusted p-value < 0.05, 28 and 61 miRNAs were significantly increased and decreased, respectively, in EBOV-infected cells. Filtering stringently for adjusted p-value < 0.001 and log2 fold-change > 1, 13 and 2 miRNAs were significantly increased and decreased (Table 1). EBOV-miR-1-5p, EBOV-miR-T1-3p, EBOV-miR-T1-5p and EBOV-miR-T3-5p/T4-5p were identified in the infected human RPE cells (Additional file 4).

**Table 1:** List of miRNAs that were differentially expressed between EBOV- and mock-infected human RPE cells at 24 h post-infection

miRNA	log2 fold-change	Adjusted p-value
hsa-miR-3074-3p	− 1.52	1.24 × 10^−12^
hsa-miR-27b-5p	− 1.06	2.63 × 10^−13^
hsa-miR-101-5p	1.03	3.09 × 10^−8^
hsa-miR-33b-5p	1.04	8.18 × 10^−5^
hsa-miR-190a-3p	1.06	6.30 × 10^−5^
hsa-miR-1305	1.10	3.92 × 10^−6^
hsa-miR-130a-5p	1.19	3.15 × 10^−5^
hsa-miR-32-5p	1.21	2.39 × 10^−6^
hsa-miR-365a-5p	1.24	2.39 × 10^−6^
hsa-miR-100-3p	1.27	1.19 × 10^−13^
hsa-miR-33b-3p	1.33	7.05 × 10^−10^
hsa-miR-4521	1.37	2.63E−13
hsa-miR-33a-5p	1.41	5.10E−04
hsa-miR-29b-3p	1.47	2.24E−08
hsa-miR-1307-5p	1.50	1.98E−10

**Fig. 1:**
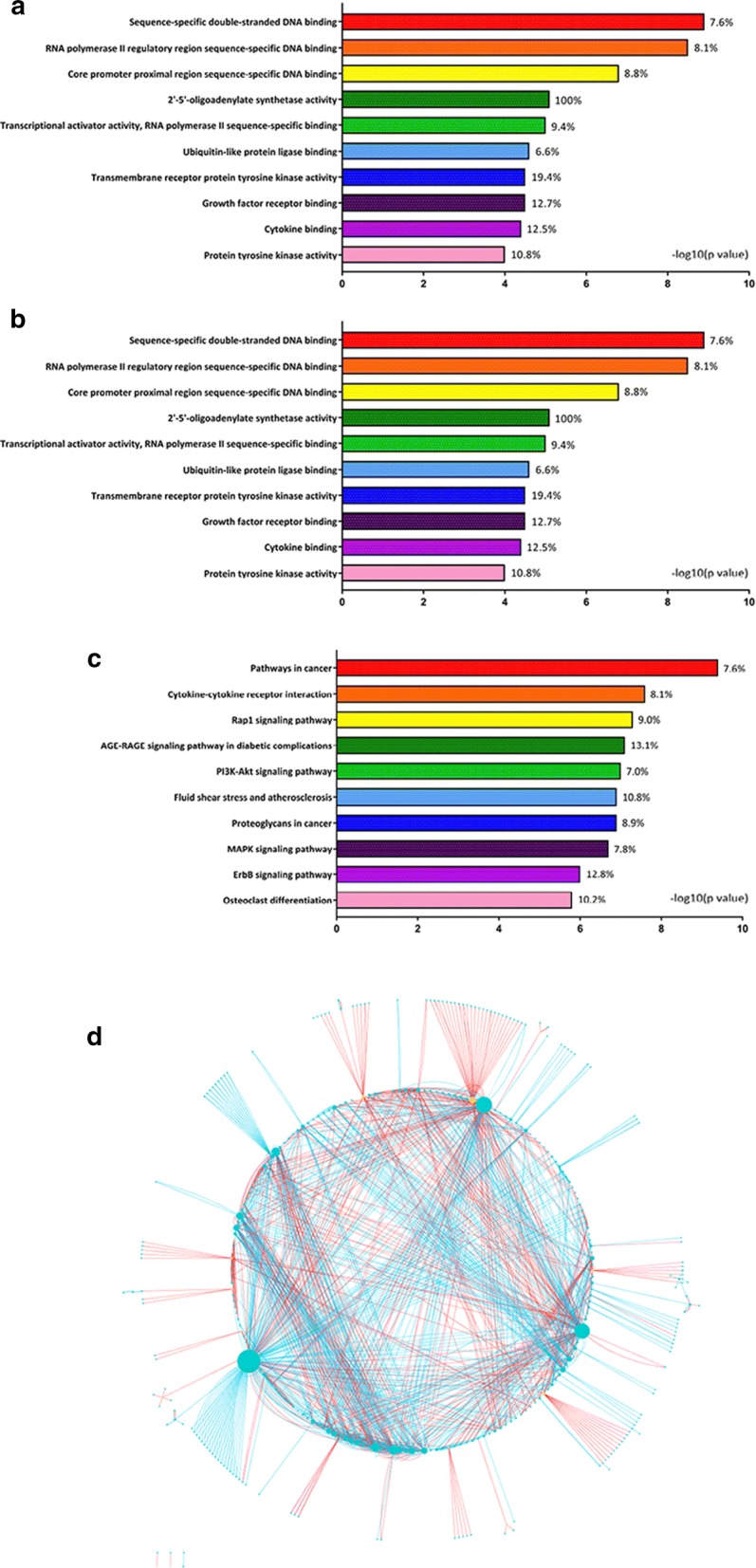
**a**–**c** Graphs showing enriched gene ontology categories: **a** biological process, **b** molecular function and **c** KEGG pathways in human RPE cells 24 h post-infection with EBOV. Percentages indicate proportion of known genes represented within the grouping. **d** Network of interactions between protein-coding genes and miRNAs in EBOV-infected human RPE cells, with miRNAs indicated by yellow diamonds, molecules indicated by blue circles, and interactions that are promoting or inhibiting represented as blue or red lines, respectively. Size of circle or diamond is proportionate to the number of molecular interactions (or degrees). An enlarged version of **d** is presented as Additional file 8

Putative target genes in EBOV-infected human RPE cells were predicted from the 15 highly differentially expressed miRNAs using algorithms: 2629 targets by Diana microT; 1799 targets by miRDB; and 14,315 targets by TargetScan (Additional file 5). Targets were also identified through interrogation of experimentally validated data: 44 targets by miRecords; and 9 targets by miRTarBase (Additional file 6). 1440 genes were differentially expressed between EBOV- and mock-infected ARPE-19 cells (Additional file 7). Highly enriched gene ontology categories and KEGG pathways were established for the target genes, based on numbers represented within categories and pathways, and ranking of corrected p-values (Fig. 1a–c, Additional file 8). The molecular network constructed from miRNA-target gene interactions included 363 highly connected molecules; most connected molecule was leucine-rich repeat kinase 2 (LRRK2), and most connected miRNA was hsa-miR-190a (Fig. 1d, Table 2, Additional files 9 and 10).

**Table 2:** List of highly-connected protein-coding genes (16 most connected) and miRNAs (all connected in EBOV-infected human RPE cells)

Protein-coding genes	Degree
LRRK2	181
MAPK13	125
MAPK7	117
PRKCE	66
PRKCH	64
SGK494	64
RND3	52
DIRAS3	44
RASD2	43
RASL11B	43
PDGFRB	37
ARHGEF3	36
IQGAP2	36
ARL14	35
ARL4A	35

miRNA	Degree
hsa-miR-190a	58
hsa-miR-29b-3p	25
hsa-miR-130a-5p	17
hsa-miR-32-5p	16
hsa-miR-33a-5p	12
hsa-miR-33b-5p	11
hsa-miR-7-5p	11
hsa-miR-33b-3p	7
hsa-miR-1305	6
hsa-miR-100-3p	5
hsa-miR-19a-5p	5
hsa-miR-101-5p	4
hsa-miR-27b-5p	3
hsa-miR-365a-5p	1
hsa-miR-4521	1

Degree indicates number of interactions for each molecule

### Discussion

*Zaire ebolavirus* causes uveitis in EVD survivors [26, 27]. We have identified miRNAs produced in human RPE cells following infection with EBOV. Viruses take advantage of host miRNAs to adjust gene expression and create an environment that supports replication [28]. Human RPE cells make multiple molecular adjustments in response to infection with EBOV [5], and miR-190a is central to the cell response. This highly connected miRNA contributes to cell survival and latency in Epstein–Barr virus (EBV) infection, by downregulating expression of tumor protein P53 inducible nuclear protein 1 and nuclear receptor subfamily 4 group A member 3, thereby preventing cell cycle arrest and inhibiting apoptosis [29]. The same mechanism may be utilized by EBOV to promote viral replication in infected RPE cells. One other interacting miRNA—miR-101—prevents cell death in the context of herpes simplex virus type 1 infection [30], pointing to a common strategy among viruses to promote survival of both the virus and its host cell.

Another highly connected miRNA was miR-29b3p, which suppresses key immunological pathways by downregulating nuclear factor kappa-light-chain-enhancer of activated B cells (NF-κB) [31]. This transcription factor plays an essential role in the anti-viral type IFN response. Multiple receptors—including toll-like receptors and retinoic acid-inducible gene (RIG)-I—stimulate NF-κB, which activates transcription of IFN-β and IFN-stimulated genes. *Zaire ebolavirus* profoundly inhibits the type I IFN response in monocytes and other cell populations, through the action of viral proteins—VP24 and VP35—and suppression of RIG-I signaling [32]. Our prior work has demonstrated that EBOV-infected RPE cells mount a type I IFN response [5]; however, EBOV also may suppress this pathway through activation of miR-29b-3p. Other viruses act similarly: the NF-κB pathway is not activated in human RPE cells infected with cytomegalovirus [33]. MicroRNA-29b is also activated in Japanese encephalitis virus infection, particularly in persons with neurological sequelae, and elevated serum levels may indicate severe disease [34].

After entering the host cell, EBOV is transported via endolysosomal trafficking [35]. Infected cells also release exosomes containing EBOV VP40, which may promote leukocyte apoptosis [36]. Consistent with involvement of the endolysosomal system, the most highly connected molecule in the miRNA-target gene network was LRRK2. This protein is a key regulator of the system; a range of mutations in LRRK2 result in formation of large, dysfunctional lysosomes [37]. Interestingly, LRRK2 activation in microglia has been implicated in the progression of central nervous system inflammation, as occurs in Parkinson’s disease and HIV-associated neurocognitive disorders [38]. This suggests LRRK2 activity in EBOV-infected RPE cells may contribute to the development of uveitis.

As applies to all viruses, EBOV infection involves hijacking translational machinery of the host cell to produce viral particles. Gene ontology enrichment analysis for molecular function showed strong activation of DNA-binding and transcription pathways in EBOV-infected human RPE cells. Known to bind double-stranded RNA, multiple genes in the 2′-5′-oligoadenylate synthetase pathway were upregulated in our gene ontology enrichment analyses, indicating a miRNA-facilitated host response to EBOV. Activation of kinases was prominent; pathways involving kinases—such as protein kinase R—are directly involved in the response to EBOV, binding to double-stranded RNA, activating cellular targets and inhibiting the host translational machinery [39].

Virus-infected cells release exosomes containing host- and virus-derived miRNAs that modulate gene expression in uninfected neighbouring cells [40]. Human RPE cells have the capacity to secrete exosomes containing miRNAs [41]. Our data indicate that hsa-miR-27b-5p and hsa-miR-3074-3p are expressed at reduced levels when the cells become infected with EBOV, which might indicate active export of the miRNAs into exosomes. Both miRNAs are detected in the aqueous humour of healthy eyes, at levels higher than found in plasma indicating local production [42]. We speculate that levels of these miRNAs might be increased in the ocular fluid of EBOV survivors, particularly those with uveitis and present biomarkers for ocular involvement. Other investigators have identified a panel of 8 serum miRNAs that predict pre-symptomatic EBOV infection, facilitating an early diagnosis of EVD [43].

RNA viruses do not typically encode miRNAs [28]. However, our analysis of EBOV-infected RPE cells identified EBOV-miR-1-5p, which has been described by several investigator teams [13, 44]. This ortholog of human miR-155 inhibits expression of importin-α5 and impacts type I IFN signaling [45]. Molecular mimicry of miR-155 is also seen in Kaposi sarcoma herpesvirus infection of B-cells, enabling the virus to drive the cell to a state that supports long-term latency and avoids apoptosis [46]. Several other EBOV-derived miRNAs were identified in the infected RPE cells, but with very low read counts suggesting negligible biological activity.

### Conclusion

Our work provides new information about the potential post-transcriptional regulation of the human RPE cell response to infection with EBOV. Review of the biological targets of the 15 highly-induced or repressed miRNAs indicates a broad range of potential regulatory activities, including effects on immune responses, cellular metabolism, cell cycle progression, apoptosis and autophagy in the host cells. MicroRNA expression varies by tissue, including within the eye [47, 48]; hence similar changes may not occur in other ocular tissues or tissues from other immune-privileged sites, such as testis. Future studies of the regulatory activities of these miRNAs in human RPE cells should delineate their involvement in the intraocular persistence of EBOV and EBOV-associated uveitis in EVD survivors.

## Limitations

- We studied EBOV infection in the ARPE-19 human RPE cell line, in place of cells isolated from human eyes. This cell line is a well-characterized, robust model for studying human retinal pigment epithelium [5], and thus our findings should provide reasonable fidelity to an ocular infection in an EVD survivor.
- MicroRNA expression was evaluated at one time-point post-infection. The time-point was selected in our previously published study [7], to be maximally informative of host cell responses to EBOV infection, and may be most applicable to EVD and early survivorship.
- Human RPE cells were studied in isolation, while the intraocular environment includes multiple cell populations. However, this approach allowed us to focus specifically on gene expression in a key intraocular target cell for EBOV.
- Cells were infected with virulent EBOV, which caused recent EVD epidemics; less virulent *Ebolavirus* species may induce different miRNA expression. Cells were not co-infected with other pathogens: co-infection influences EVD outcome, which might in part reflect altered miRNA expression, as demonstrated in other infections [49, 50].

## Supplementary information

**Additional file 1.** Trimming statistics for RNA sequencing data generated for small RNA expressed in human RPE cells at 24 h following infection with EBOV or mock-infection.

**Additional file 2.** Lists of total and differentially expressed host miRNAs expressed in human RPE cells at 24 h following infection with EBOV or mock-infection.

**Additional file 3.** Graphic showing multidimensional scaling of small RNA expressed in human RPE cell at 24 h post-infection with EBOV.

**Additional file 4.** List of EBOV miRNAs expressed in human RPE cells at 24 h following infection with EBOV or mock-infection.

**Additional file 5.** List of predicted miRNA targets from Diana microT, miRDB and TargetScan databases.

**Additional file 6.** List of validated miRNA targets from miRecords and miRTarBase databases.

**Additional file 7.** List of differentially-expressed genes in human RPE cells at 24 h following infection with EBOV or mock-infection.

**Additional file 8.** List of enriched gene ontology categories (biological process, molecular function) and KEGG pathways for biological targets of differentially expressed miRNAs in EBOV- versus mock-infected human RPE cells.

**Additional file 9.** Results of network construction based around differentially expressed miRNAs in EBOV- versus mock-infected human RPE cells.

**Additional file 10.** Enlargement of Fig. 1d.

## Data Availability

All data generated or analysed during this study are included in this published article and its supplementary information files, with the exception of raw data files, which have been lodged in the Gene Expression Omnibus (GEO) of the National Centre for Biotechnology Information (NCBI) as GSE136985.

## Abbreviations

cDNA: complementary DNA
DNA: deoxyribonucleic acid
EBOV: *Zaire ebolavirus*
EBV: Epstein–Barr virus
EVD: Ebola virus disease
IFN: interferon
KEGG: Kyoto Encyclopedia of Genes and Genomes
LRRK2: leucine-rich repeat kinase 2
mRNA: messenger RNA
miRNA: microRNA
NF-κB: nuclear factor kappa-light-chain-enhancer of activated B cells
Nt: nucleotide
RIG-I: retinoic acid-inducible gene I
RNA: ribonucleic acid
RNA-Seq: RNA sequencing
RPE: retinal pigment epithelial
