# Supplementary material for: Expression of microRNA in human retinal pigment epithelial cells following infection with Zaire ebolavirus
Source: BMC Res Notes. 2019 Oct 1;12:639. doi: 10.1186/s13104-019-4671-8 (PMC6771106; doi:10.1186/s13104-019-4671-8)
Supplement: Supplementary file 1 — Additional file 1. Trimming statistics for RNA sequencing data generated for small RNA expressed in human RPE cells at 24 h following infection with EBOV or mock-infection. [file 13104_2019_4671_MOESM1_ESM.docx]

**Additional File 1: Trimming statistics.** Trimming of RNA sequencing data on small RNA extracted from human RPE cells at 24 hours after infection with EBOV.

| Isolate | Total reads processed | Reads with adapters | Long reads | Reads written (passing filters) |
| --- | --- | --- | --- | --- |
| EBOV 1 | 2.87 x 10^7^ | 2.83 x 10^7^ (98.6%) | 1.51 x 10^6^ (5.3%) | 2.72 x 10^7^ (94.7%) |
| EBOV 2 | 1.61 x 10^7^ | 1.57 x 10^7^ (97.6%) | 0.50 x 10^6^ (3.1%) | 1.56 x 10^7^ (96.9%) |
| EBOV 3 | 3.88 x 10^7^ | 3.81 x 10^7^ (98.2%) | 1.30 x 10^6^ (3.4%) | 3.75 x 10^7^ (96.6%) |
| Mock 1 | 2.14 x 10^7^ | 2.05 x 10^7^ (95.7%) | 0.94 x 10^6^ (4.4%) | 2.05 x 10^7^ (95.6%) |
| Mock 2 | 1.95 x 10^7^ | 1.92 x 10^7^ (98.7%) | 1.42 x 10^6^ (7.3%) | 1.81 x 10^7^ (92.7%) |
| Mock 3 | 2.30 x 10^7^ | 2.26 x 10^7^ (98.1%) | 2.03 x 10^6^ (8.8%) | 2.10 x 10^7^ (91.2%) |
