# Supplementary figures and images for: Expression of microRNA in human retinal pigment epithelial cells following infection with Zaire ebolavirus
Source: BMC Res Notes. 2019 Oct 1;12:639. doi: 10.1186/s13104-019-4671-8 (PMC6771106; doi:10.1186/s13104-019-4671-8)

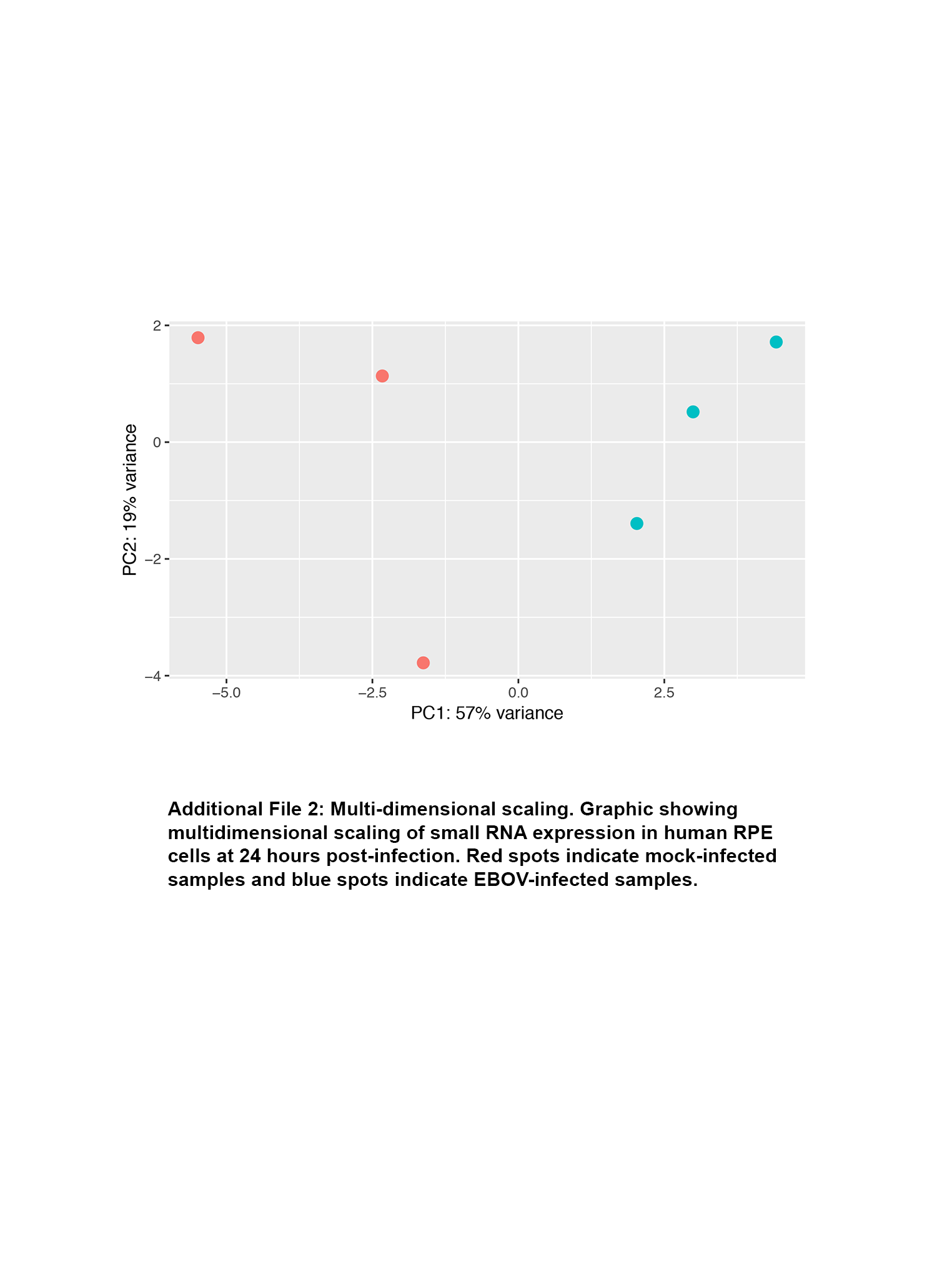

Supplement: Supplementary file 3 — Additional file 3. Graphic showing multidimensional scaling of small RNA expressed in human RPE cell at 24 h post-infection with EBOV. [file 13104_2019_4671_MOESM3_ESM.tif]

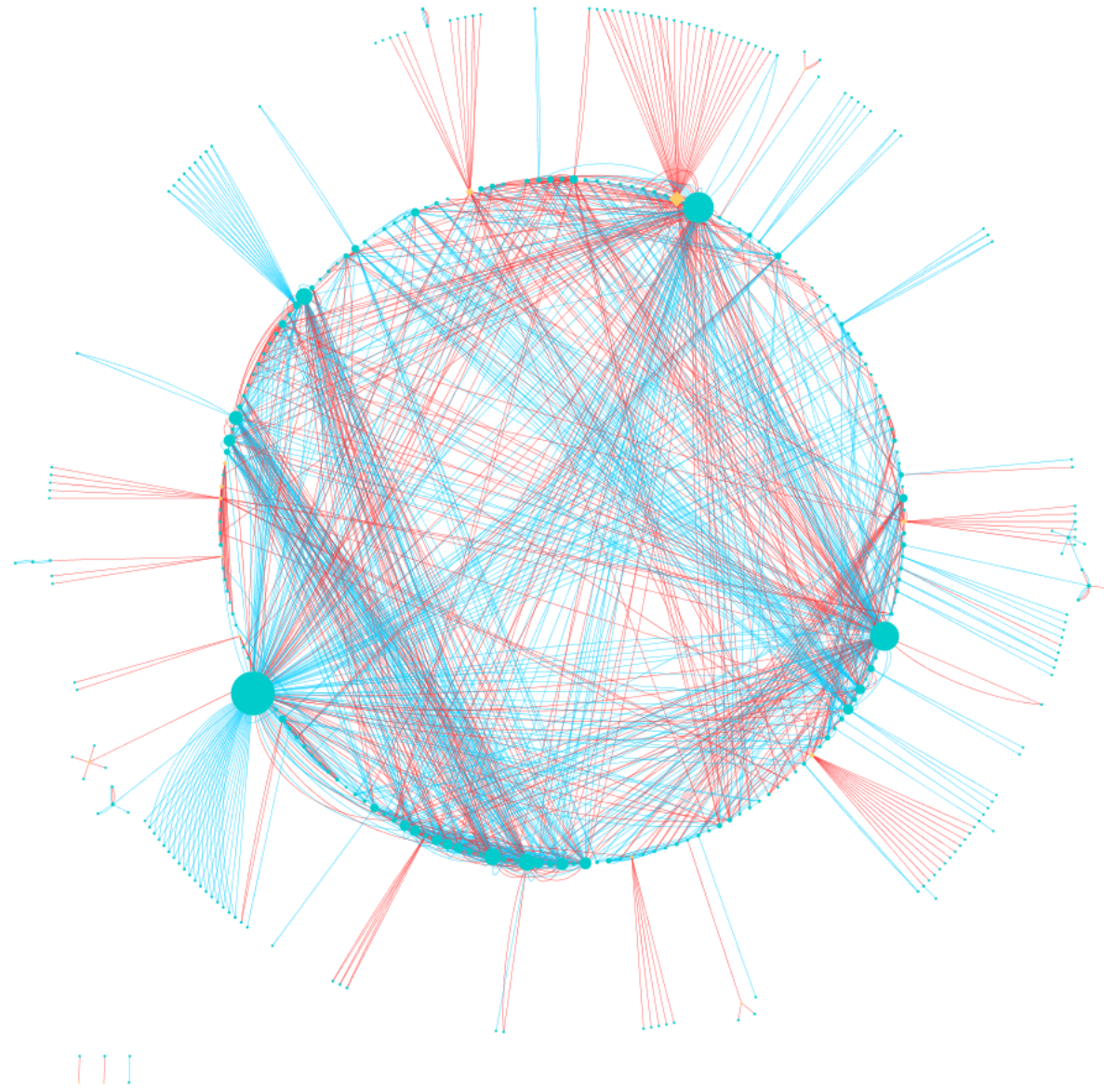

Supplement: Supplementary file 10 — Additional file 10. Enlargement of Fig. 1d. [file 13104_2019_4671_MOESM10_ESM.pdf]
